# Higher emissions scenarios lead to more extreme flooding in the United States

**DOI:** 10.1038/s41467-023-44415-4

**Published:** 2024-01-03

**Authors:** Hanbeen Kim, Gabriele Villarini

**Affiliations:** 1 IIHR—Hydroscience and Engineering, University of Iowa, Iowa City, USA; 2 Present Address: Department of Civil and Environmental Engineering, Princeton University, Princeton, USA; 3 Present Address: High Meadows Environmental Institute, Princeton University, Princeton, USA

**Keywords:** Projection and prediction, Hydrology, Water resources

## Abstract

Understanding projected changes in flooding across the contiguous United States (CONUS) helps increase our capability to adapt to and mitigate against this hazard. Here, we assess future changes in flooding across CONUS using outputs from 28 global climate models and four scenarios of the Coupled Model Intercomparison Project Phase 6. We find that CONUS is projected to experience an overall increase in flooding, especially under higher emission scenarios; there are subregional differences, with the Northeast and Southeast (Great Plains of the North and Southwest) showing higher tendency towards increasing (decreasing) flooding due to changes in flood processes at the seasonal scale. Moreover, even though trends may not be detected in the historical period, these projected future trends highlight the current needs for incorporating climate change in the future infrastructure designs and management of the water resources.

## Abstract (web summary)

This paper assesses future changes in flood magnitude across the conterminous United States based on multiple climate change scenarios. The results suggest that annual maximum peak discharge is projected to become more extreme under higher emission scenarios.

## Introduction

Much of the existing infrastructure and water resources management are based on the assumption of stationarity, implying that the statistical properties observed in the past will persist into the future. While stationarity has been the cornerstone of most of the flood frequency studies aiming at water resources management and infrastructure design, this working assumption has been challenged due to changes in the climate system and in the way we manage our landscapes^1^. However, this signal of change has been hard to find in observational studies due to the variability in the flood records (i.e., small signal-to-noise ratio), complexity in the physical processes leading to flooding, and large uncertainties^2,3^. The Intergovernmental Panel on Climate Change (IPCC) states that “confidence about peak flow trends over the past decades on the global scale is low”^3^ even though there are areas, such as the northeastern and southwestern United States, that have been experiencing increasing and decreasing trends^4^, respectively.

While the detection of changes is scientifically interesting, it provides limited insight for the design of future infrastructures^5^. Much effort has been placed in detecting trends in the observed floods series^6–13^; however, the only way to extrapolate these findings into the future is by assuming that the detected trends will persist in the decades to come^14,15^. Moreover, if we do not detect a trend in the historical past, are we sure that it is not going to manifest itself in the future? Because of these issues, a much more robust approach for the design of future water-related structures and projects includes first an analysis of the historical discharge records not from the perspective of the detection of changes, but rather of the attribution of the major drivers responsible for the observed interannual variability^10,16,17^. By looking back at the observational records and understanding of how different mechanisms (e.g., rainfall, snowmelt) were responsible for flooding, we can then move forward by examining how these drivers are projected to change and their implications in terms of changes in flood magnitudes.

There are two broad approaches for the attribution of nonstationarity in flood magnitudes^18^, simulation- and regression-based approaches. The former has the advantage of the use of mathematical equations describing the physical processes in flood discharge^19^, despite a greater computational burden. On the other hands, the regression-based approach models the statistical relationships between flood discharge and its possible drivers such as climate variables^20^, large-scale climate indices^21^, and land cover^22^. The main advantages of the statistical models are the computational cost and the flexibility in incorporating different process-related predictors, making these models competitive with the simulation-based approach in terms of model performance^23^. Moreover, they allow for a probabilistic view of discharge, enabling the assessment of changes in different parts of the flood peak distribution. Many of the published studies about projected changes in floods across the contiguous United States (CONUS) tend to follow a simulation-based approach at the regional or basin scales^24–29^, while only a few studies used a regression-based approach for hundreds of streamgages distributed across CONUS^23,30^.

Here, we assess future changes in flood magnitude across CONUS by taking advantage of the strengths and skill by the regression-based approach and the dense network of thousands of streamgages by the U.S. Geological Survey (USGS). We build on the statistical modeling framework described in ref. ^17^, which allows the attribution of the observed interannual variability in the annual maximum discharge records to basin- and season-averaged precipitation and temperature (see Methods). These variables are available from many global climate models (GCMs) and were shown to drive the year-to-year changes in annual maximum flood peaks across large areas of CONUS. By projecting precipitation and temperature and using them as inputs for the selected statistical models^17^, we quantify the projected changes in flood hazard over the 21st century across multiple Shared Socioeconomic Pathways (SSPs) (see Methods). Besides its good performance in reproducing historical observations, we have selected this approach because it allows us to consider the projected changes in flooding for any annual exceedance probabilities (AEPs), providing a framework for nonstationary flood frequency analyses under different scenarios.

## Results and discussion

As a preliminary step, we check whether the GCM outputs can reproduce what was observed during the historical period. We evaluate the suitability of basin- and season-averaged GCM outputs in reproducing the observed trends in annual maximum discharge (see Methods). Based on the results in Supplementary Fig. 1, there are 28 GCMs that were able to satisfactorily reproduce the observed trends over the historical period, and those are the ones we use for the projections.

### Projected changes in flood extremes across CONUS

**Fig. 1:**
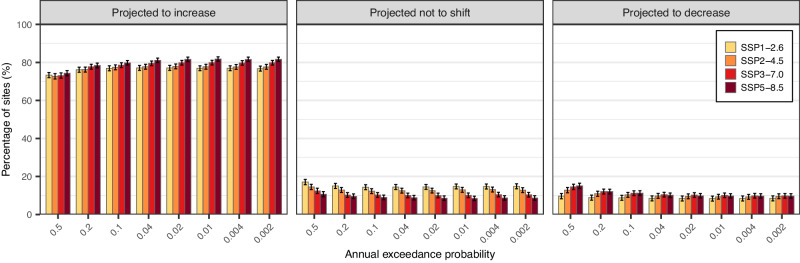
Projected shifts in the distribution of the annual maximum discharge during the historical (1985–2014) and future (2071–2100) periods for CONUS. The left (right) panel shows the percentage of sites with a significant increasing (decreasing) shift in peak discharges for CONUS at the 5% level. The middle panel shows the percentage of sites where there is no significant shift in peak discharges at the 5% level. In each panel, redder bars indicate higher emission scenarios from SSP1-2.6 to SSP 5-8.5. The error bars represent the 95% confidence intervals for multinomial proportions computed based on the Sison-Glaz method^62^.

To assess future changes in flood hazard across scenarios, we test whether the AEP distribution tends to shift towards larger or smaller values compared to the past (i.e., 2071-2100 vs. 1985-2014) under four SSPs (Fig. 1). Across CONUS, there is a general shift towards larger extremes regardless of scenarios: for a 0.5 AEP (i.e., it corresponds to a 2-year event), there are 72.7%–74.3% of the sites showing a positive shift (i.e., the distribution of the 0.5-AEP discharge is projected to shift towards larger discharge values), with this number increasing to 76.7%–81.6% for the 0.002 AEP (i.e., it corresponds to a 500-year event). On the other hand, the negative shifts between the historical period and the last 30 years of the 21st century (i.e., the distribution is projected to shift towards smaller discharge values) are limited to ~8.3%–15.1% of the sites, while a lack of detectable shifts is limited to ~8.5%–17.0% of the records depending on the AEP. Overall, these results indicate that annual maximum peak discharge is projected to become more extreme, with a larger tendency towards larger peaks. This is particularly true as we move from smaller to larger flood peaks (i.e., from the 0.5 to the 0.002 AEPs).

There is also a dependence in terms of emission scenarios (Fig. 1). Under SSP1-2.6, the percentage of sites showing a positive shift increases 3.8% (from 73.3% to 77.1%) as AEP decreases (from 0.5 to 0.002). As we move from SSP1-2.6 to SSP5-8.5, the changing rate of the sites with a positive shift also increases (i.e., 5.2%, 6.8%, and 7.5% under SSP2-4.5, SSP3-7.0, and SSP5-8.5, respectively), indicating that, the higher the emission scenario, the more the flood hazard is projected to increase for the more extreme events. These changes are compensated by the changes in those sites that did not exhibit statistically significant shifts. Based on these results, efforts to reduce greenhouse gas emissions and towards sustainability are projected to decrease the flood hazard, especially for the more extreme events.

**Fig. 2:**
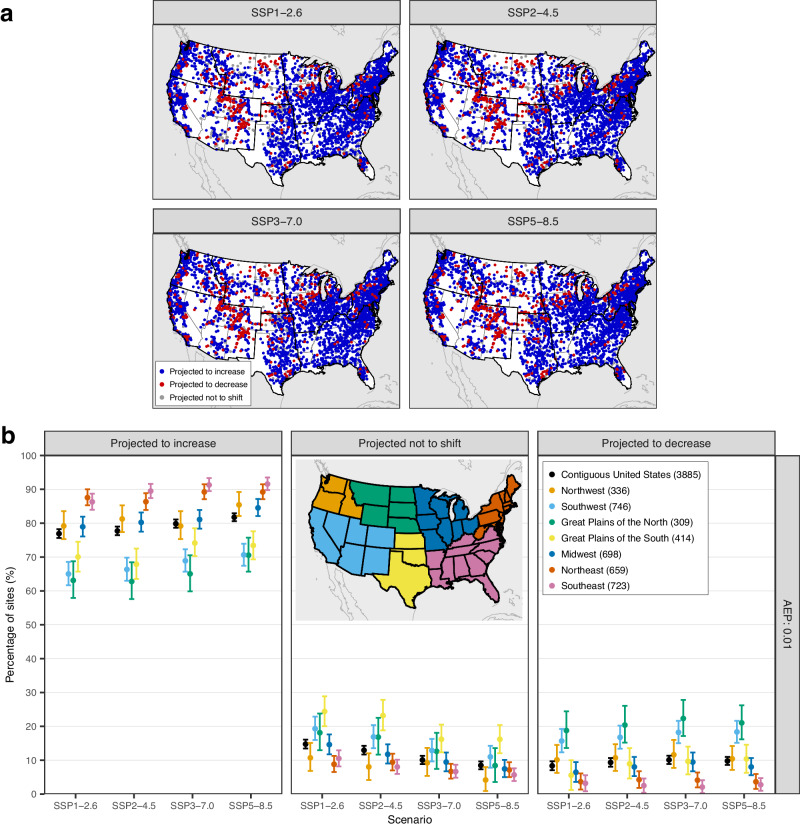
Projected shifts in the distribution of the 0.01-AEP discharge during the historical (1985–2014) and future (2071–2100) periods for CONUS and seven subregions. **a** Blue (red) circles indicate the sites with a significant increasing (decreasing) shift in peak discharges, while the gray circles indicate the sites with no significant shift at the 5% level. Bold lines represent the boundaries of seven subregions. **b** The left (right) panel shows the percentage of sites with a 5%-significant increasing (decreasing) shift in peak discharges for CONUS and its seven subregions (see inset map for the definition of subregions). The middle panel shows the percentage of sites where there is no significant shift in peak discharges at the 5% level. The error bars represent the 95% confidence intervals for multinomial proportions computed based on the Sison-Glaz method^62^. The numbers in the legend represent the number of streamgages within each region. Notice that jitters are added to the x-axis to improve readability.

If we stratify the results across the seven subregions by the U.S. National Climate Assessment (Fig. 2), different parts of the country are expected to respond differently to climate change. Flooding is projected to increase in the northeastern and southeastern United States, with more than 86% of the streamgages in that region for which the distribution of the 0.01-AEP discharge is projected to shift towards larger values. The Northwest and Midwest also exhibit shifts in the distribution towards higher values (at more than 78% of the streamgages) compared to CONUS. The picture is different for the Southwest and the Great Plains, where the percentage of sites projected to increase is below 75%, and those projected to decrease ~16%. Moreover, these regions show more of a balance between increasing and decreasing flood hazards for larger AEPs (Supplementary Fig. 2).

Our results illustrate that CONUS is projected to experience an overall increase in extreme flooding, even though the magnitude of this signal is not spatially homogeneous but varies sub-regionally. These findings expand on the recent literature on the projected changes in discharge for river basins within various subregions of the United States^24–29^, as well as CONUS^30–32^. Compared to existing studies, some of the strengths of these results are the large sample of streamgage stations, the use of CMIP6 GCM outputs, and multiple scenarios. By employing a statistical approach, we are able to project changes across different parts of the flood peak distributions, allowing accounting for the nonstationarities across different AEPs at any point in time during the 21st century. Moreover, by modeling the flood peak records by accounting for processes that are dominant during each of the four seasons (see Methods), we are moving closer to a process-driven flood frequency analysis, which is different from other regression-based modeling studies that model directly the annual maximum discharges^23,30,33^.

### Role of seasonal climate drivers responsible for peak discharge

To shed some light on the possible physical mechanisms responsible for the detected shifts and their subregional variability, we examine the projected changes in seasonal precipitation and temperature (see Methods). All GCMs project increases in temperature with respect to the historical past, regardless of seasons, with the largest shifts as we move from SSP1-2.6 to SSP5-8.5 (Supplementary Fig. 3). On the other hand, precipitation exhibits a much more variable pattern of change in both seasonally and sub-regionally (Supplementary Fig. 4). In the Northwest, Great Plains of the North, and Midwest, there is an increasing shift in winter and spring precipitation, while there is a decreasing shift in summer precipitation at most sites, with a larger signal of change for higher emission scenarios. The Southwest shows a different seasonality, with a decreasing shift in spring precipitation and an increasing shift in winter precipitation. The Northeast and Southeast show an increasing shift in precipitation for all seasons and scenarios, except for few sites in the Southeast with a projected decreasing precipitation in the summer. One commonality among regions is the exacerbation of seasonal precipitation for increasing emissions: not only the absolute value of the projected changes in precipitation is expected to increase, but also its variability among the different sites and pointing to potentially larger climate swings within these regions.

**Fig. 3:**
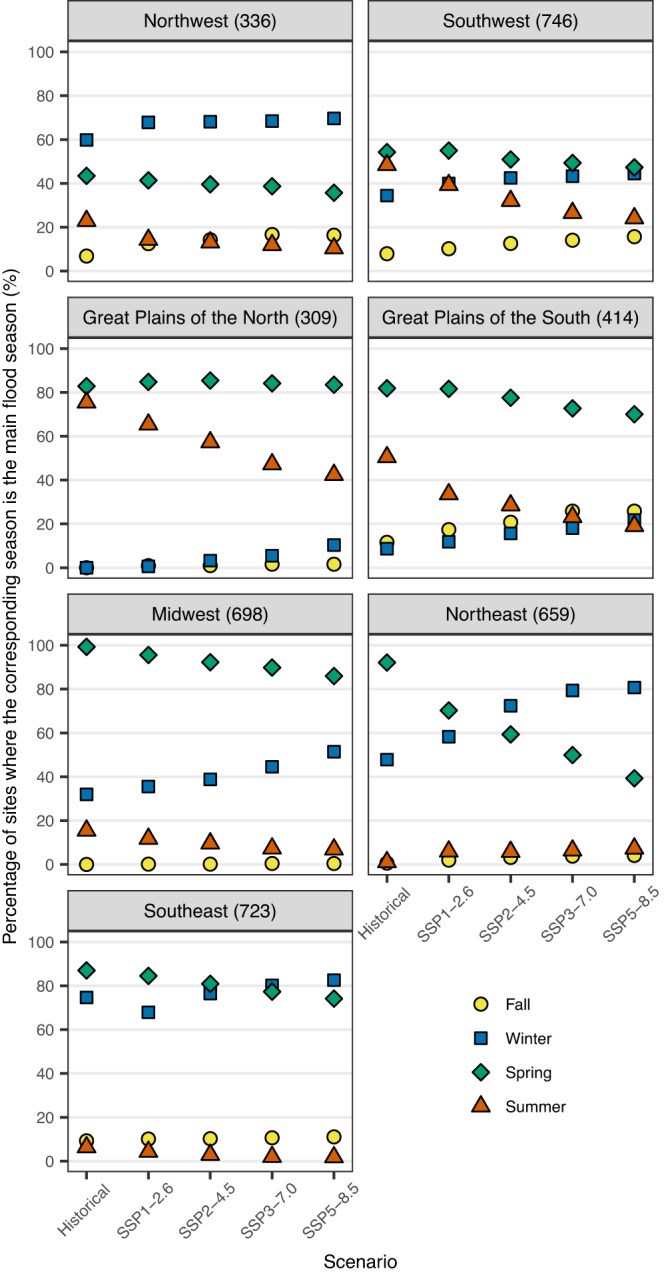
Projected changes in the main flood seasons during the historical (1985–2014) and future (2071–2100) periods for seven subregions. At each streamgage, seasons that account for at least 30% of annual maximum discharge occurrence are defined as the main flood seasons. The numbers in parentheses in the title of each panel represent the number of streamgages within each region.

To better understand the role of seasonal climate drivers in driving flood extremes, we investigate the seasonality of annual maximum discharge occurrence, given the good performance of our modeling framework in reproducing the historical flood seasonality (Supplementary Fig. 5). The seasonality of annual maximum discharge is projected to change, especially for increasing emissions, with differences depending on subregions (Supplementary Figs. 6, 7). If we focus on the main flood seasons, there is a distinct change in the seasonality of flooding across each of the seven subregions (Fig. 3). In the Northeast and Southeast, winter and spring are the main flood seasons, with winter being the dominant season for the higher emission scenarios. Therefore, the projected shift towards larger flood peaks can be interpreted through an increase in winter and spring precipitation^28^ (Supplementary Fig. 4). The winter and spring seasons are also the main flood seasons in the Northwest and Midwest, respectively, regardless of emission scenarios. The increasing shift in precipitation in those seasons (Supplementary Fig. 4) is consistent with the projected tendency towards increasing trends in flood peaks. In the Midwest, while spring is the dominant season, there is an increase in the number of sites for which winter’s contribution is projected to increase, especially for higher emissions, possibly due to the polarward shift of the tracks of extratropical cyclones^34,35^ (Supplementary Fig. 8). In the Southwest, the summer season, one of the main flood seasons in this area, is projected to contribute less to the annual maxima, particularly in the mountainous area such as the Rockies (Supplementary Fig. 7). Since snowmelt is the main flood process in this area^36,37^, spring precipitation and temperature play a crucial role in the magnitude of summer flood peaks^17^. Therefore, the decreasing (increasing) shifts in spring precipitation (temperature) can lead to the negative shifts in annual maximum discharge in this area (Fig. 2a). On the other hand, the positive shift in annual maximum discharge is likely associated with increases in precipitation in winter, the main flood season in the West Coast driven by atmospheric rivers^38^ (Supplementary Fig. 6). In the Great Plain of the North where snowmelt is the main driver of runoff potential^39^, spring and summer seasons are the main flood seasons^40^ (as also shown in Fig. 3). In addition to decreases in summer precipitation (Supplementary Fig. 4), the increases in temperature (Supplementary Fig. 3) can lead to negative shifts in annual maximum discharge by leading to snowpack decline^41^ and more rainfall than snow^42^. The Great Plain of the South shows the dominance of spring season for all scenarios. However, there is no consistency in the projected shifts in spring precipitation (Supplementary Fig. 4), consistent with the much larger degree of variability in the shift in flood extremes.

### Projected changes in trends in flood extremes

**Fig. 4:**
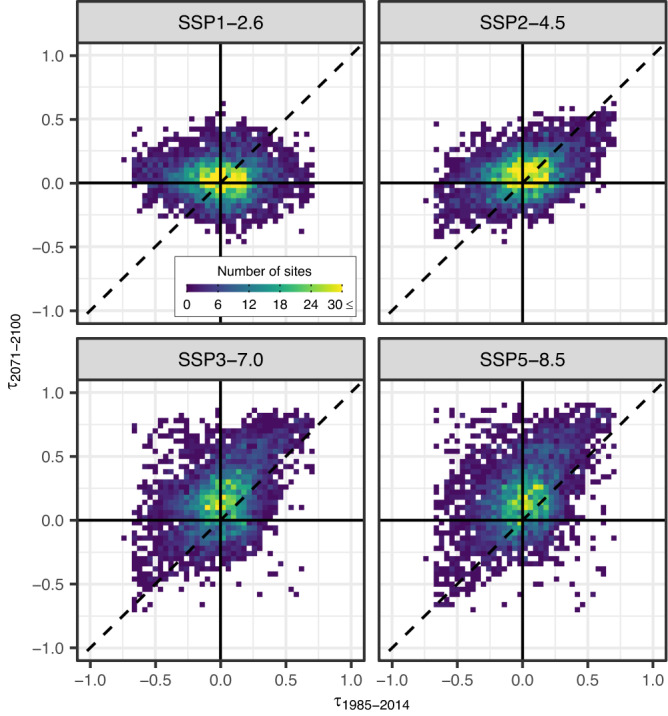
2D histogram of trends in 0.01-AEP discharge during the historical past and the future period. The Kendall’s τ is computed for the historical (1985–2014) and future (2071–2100) periods for the ensemble mean of 0.01-AEP discharge.

Here we examine whether the historical trends in annual maximum discharge are expected to persist or not in the future. There is a significant change between historical and future trends depending on scenarios (Fig. 4). Under SSP1-2.6, trends in 0.01-AEP discharge are projected to remain not significant and/or to become smaller/more negative during the 2071–2100 period compared to 1985–2014. As we consider scenarios with higher emissions, however, trends are projected to be exacerbated over the future period, including trend-sign reversal from negative/not-significant to positive in the last 30 years of the 21st century compared to the historical past. This tendency is more intensified for larger flood peaks (i.e., smaller AEPs) (Supplementary Figs. 9, 10). The results are consistent with other studies whose results are derived from hydrological models^43,44^, reinforcing the message against extrapolating the observed historical trends in flood extremes directly to project future changes in the magnitude of extreme flooding. Likewise, the lack of statistically significant trends over the historical period should not be used as a justification for not taking climate change into account^5^.

### Dependence of projected flood hazards on human-modified basins

The abovementioned results are based on 3,885 streamgages, regardless of the degree of anthropogenic modification that they experienced. To provide further evidence of the robustness of these findings as representative of a climate change signal, we stratified the basins into 752 reference and 2,989 non-reference sites according to the Geospatial Attributes of Gages for Evaluating Streamflow version II (GAGES-II) classification^45^. A reference site is defined as the least-disturbed watershed by human influences, while a non-reference site is identified as disturbed by anthropogenic activities (e.g., reservoirs and dams). Based on Supplementary Figs. 11–13, the results for the two classes of basins are similar (compare them to Figs. 1, 2 and 4 as well), pointing to the robustness of our results.

One way to interpret these results is that the most influential factor for extreme outflows of hydraulic structures is extreme inflow^46^, which in turn could be dominated by climate drivers. In addition, seasonal total precipitation is less sensitive to the lag effect of hydraulic structures and can be therefore a reasonable covariate for modeling flood extremes in a regulated basin^33^. These are possible reasons for the good performance of our statistical models, despite the use of only basin- and season-averaged climate drivers and the lack of hydraulic structures as predictors^17^. Although changes in the climate system play a dominant role in driving changes in flood hazard^47^, the role of hydraulic structures in mitigating flood risk under climate change should not be overlooked^48,49^. Therefore, future work should focus on modeling the annual maximum discharge records with additional dam or reservoir parameters and comparing the results to the existing model with only climate-related parameters to allow for a more accurate assessment of the impact of anthropogenic modification on the projected flood extremes.

The Fifth National Climate Assessment highlights the challenge of flood projections under climate change^50^. This challenge arises from the intricate interplay of multiple hydrological processes within flood-generating mechanisms, making it insufficient to extrapolate historical trends in the flood series. Our findings can provide valuable and direct information towards the detection, attribution, and projection of flooding by focusing on changes in climate drivers under different emission scenarios, not on historical trends of flood extremes. The main limitation of our climate-informed projections is the assumption that the statistical relationship between flood discharge and its climate drivers does not vary over time. This relationship could be modulated by non-climate drivers (e.g., urbanization^51^ and irrigation^52^), implying that our projections of flooding are limited to responses to changes in only climate drivers and may involve additional uncertainties.

Incorporating climate change into design decisions of water-related structures and projects still remains a major challenge in engineering practice^53^. Given that multiple emission scenarios are available, considering the worst-case scenario could represent a path forward, although scenario uncertainty is relatively small compared to other factors (e.g., GCMs and climate variability)^54^. Regarding design metrics, it has been suggested to replace the traditional measure (i.e., average return period) with other design concepts such as reliability or risk of failure over a planning horizon for better communicating under nonstationary conditions^15^. Because of its probabilistic nature, we have the entire statistical distribution of annual maximum discharge for any year, which can provide discharge values for different design metrics and scenarios over the 21st century. This feature can pave the way to supporting some of the most pressing needs in a robust design of the physical infrastructure^55^.

## Methods

### Statistical modeling of annual maximum discharge

We adopt the statistical attribution approach by Ref. ^17^, which showed good performance in reproducing the annual maximum mean daily discharge time series across the CONUS. For a given location, the method does not model directly the annual maximum discharge record, but it breaks it down into four seasonal models, reflecting different flood-generating mechanisms that comprise the population of the annual maxima. Therefore, the first step is to model the seasonal maximum mean daily discharge in terms of basin- and season-averaged precipitation and temperature using gamma regression models. Then, by means of Monte Carlo simulations, the four seasonal models are mixed to obtain the derived distribution of the annual maxima. In this study, we utilize the developed gamma regression models whose parameters were estimated for 3885 streamgages across CONUS in Ref. ^17^. We also follow their Monte Carlo approach to obtain annual maximum discharge with various annual exceedance probabilities (AEPs). Here, we iterate the simulation experiments 10,000 times to obtain more reliable extreme discharge series with lower AEPs; 0.5, 0.2, 0.1, 0.04, 0.02, 0.01, 0.004, and 0.002. See Ref. ^17^ for details on the statistical attribution methodology.

### Post-processing of global climate model outputs

To assess future changes in flood extremes, we use the projected precipitation and temperature from GCMs part of the Coupled Model Intercomparison Project Phase 6 (CMIP6)^56^. We consider the GCMs that provide both precipitation (‘pr’) and near-surface air temperature (‘tas’) variables at a monthly time scale and nominal spatial resolutions of no more than 250 km. We obtain monthly mean ‘pr’ (kg m^−2^ s^−1^) and ‘tas’ (K) for 36 GCMs that have outputs under historical and four standard SSPs^57^: SSP1-2.6, SSP2-4.5, SSP3-7.0, and SSP5-8.5. The information about the 36 GCMs is summarized in Supplementary Table 1.

To use the projected variables as regression model inputs, we first convert monthly mean ‘pr’ and ‘tas’ into monthly total precipitation (mm) and monthly mean temperature (°C) and calculate the basin-averaged values based on the basin boundary data (USGS Streamgage NHDPlus Version 1 Basins 2011). We then bias-correct each GCM output and each month with respect to the Parameter-Elevation Regression on Independent Slopes Model (PRISM) data^58^ (i.e., reference data) for 1950–2014; we bias-correct the GCM outputs using empirical quantile mapping by the Santander Meteorology Group^59^. The bias-corrected monthly series are aggregated and averaged at the seasonal scale. Consequently, we obtain the basin- and season-averaged precipitation and temperature and use them as predictors for annual maximum discharge.

### Evaluation of GCMs in reproducing trends in peak discharge

We use the basin- and season-averaged drivers derived from the GCMs as inputs to the abovementioned gamma regression models. To assess the suitability of the GCMs for projections, we first evaluate their capability in reproducing the observed trends over the historical period. More specifically, we compare the trend in annual maximum discharge series obtained when using the GCMs and the PRISM over the 1951–2014 period by means of the Mann-Kendall trend test^60,61^. Depending on the test results at the 5% significance level, streamgage stations are classified into “Match” and “Mismatch” groups as follows:

- Match group: Both annual maximum discharge series derived from the GCM and PRISM have a statistically significant trend and of the same trend sign, or they are both not statistically significant.
- Mismatch group: Annual maximum discharge series derived from the GCM (or PRISM) has modeled (observed) Kendall’s τ values that are not statistically significant, while observed (modeled) Kendall’s τ values are significant. Or both annual maximum discharge series derived from the GCM and PRISM have a significant trend, but of opposite sign.

Among the 36 GCMs we analyze, we select 28 GCMs in which the percentage of sites belonging to the match group is larger than 70% (Supplementary Fig. 1). For each selected GCM, we obtain annual maximum discharge series with various AEPs (i.e., 0.5, 0.2, 0.1, 0.04, 0.02, 0.01, 0.004, and 0.002) from the simulated series. Then we use the ensemble mean of the annual maximum discharge series for the selected GCMs to investigate how the magnitude and the trend of flood extremes could change in the future.

### Detection of projected changes in flood extremes

We use a Monte Carlo approach to detect the significant increasing (or decreasing) projected changes in annual maximum discharge considering the uncertainties in the GCM ensemble mean. This simulation-based approach allows the detection of statistically significant changes in a given AEP based on the ensemble mean of the GCMs and their variability. The procedure for detecting projected changes is as follows:

1. For each year, estimate the sampling distribution parameters of the mean of the selected GCMs’ annual maximum discharge based on the central limit theorem.
2. From the sampling distribution, generate samples of the ensemble mean for historical and future periods.
3. Calculate the difference of median values between historical and future periods:
  1 $$\Delta Q=\frac{{Q}_{{med}}^{{future}}-{Q}_{{med}}^{{hist}}}{{Q}_{{med}}^{{hist}}}$$
  where ${Q}_{{med}}^{{hist}}$ and ${Q}_{{med}}^{{future}}$ are the median values of generated flood extremes for historical and future periods, respectively.
4. Iterate steps (2)–(3) 10,000 times and calculate 2.5%, 50%, and 97.5% quantiles of $\Delta Q$ (i.e., ${\Delta Q}_{0.025}$, ${\Delta Q}_{0.5}$, and ${\Delta Q}_{0.975}$).
5. Consider an increase (decrease) shift if the signs of all ${\Delta Q}_{0.025}$, ${\Delta Q}_{0.5}$, and ${\Delta Q}_{0.975}$ are positive (negative), while no shift if any signs of ${\Delta Q}_{0.025}$, ${\Delta Q}_{0.5}$, or ${\Delta Q}_{0.975}$ are different from the others.

For each site, the significant change of flood extreme for various AEPs and scenarios between historical (1985–2014) and future (2071–2100) periods is identified by conducting the simulation-based approach.

### Projected changes in precipitation and temperature

To examine what climate drivers affect the projected shifts in flood extremes, we first obtain the ensemble mean of seasonal- and basin-averaged precipitation ($P.{ens}$) and temperature ($T.{ens}$) for all scenarios. Then we calculate the projected change in $P.{ens}$ and $T.{ens}$ as follows:

2 $$\Delta P.{ens}={P.{ens}}_{{med}}^{{future}}-{P.{ens}}_{{med}}^{{hist}}$$

3 $$\Delta T.{ens}={T.{ens}}_{{med}}^{{future}}-{T.{ens}}_{{med}}^{{hist}}$$

where ${P.{ens}}_{{med}}^{{hist}}$ (${T.{ens}}_{{med}}^{{hist}}$) and ${P.{ens}}_{{med}}^{{future}}$ (${T.{ens}}_{{med}}^{{future}}$) are the median values of $P.{ens}$ ($T.{ens}$) for historical (1985–2014) and future (2071–2100) periods, respectively. For each corresponding season, we also calculate the relative change in the ensemble mean of seasonal maximum discharge ($Q.{ens}$):

4 $$\Delta Q.{ens}=\frac{{Q.{ens}}_{{med}}^{{future}}-{Q.{ens}}_{{med}}^{{hist}}}{{Q.{ens}}_{{med}}^{{hist}}}$$

where ${Q.{ens}}_{{med}}^{{hist}}$ and ${Q.{ens}}_{{med}}^{{future}}$ are the median values of $Q.{ens}$ for historical (1985–2014) and future (2071–2100) periods, respectively.

### Supplementary information

Supplementary Information

## Data Availability

The USGS basin boundary data is available from the USGS Streamgage NHDPlus Version 1 Basins 2011 at https://water.usgs.gov/lookup/getspatial?streamgagebasins. The PRISM precipitation and temperature are obtained from the PRISM climate group^58^ and available at https://prism.oregonstate.edu/historical/. The historical and future projected precipitation and temperature from the CMIP6 GCMs are available at https://esgf-node.llnl.gov/search/cmip6/. The GAGES-II data is available at https://water.usgs.gov/GIS/metadata/usgswrd/XML/gagesII_Sept2011.xml.

## Code availability

Codes that were used in this study are available upon request.
