# Author Correction: Higher emissions scenarios lead to more extreme flooding in the United States

**DOI:** 10.1038/s41467-024-48129-z

**Published:** 2024-05-01

**Authors:** Hanbeen Kim, Gabriele Villarini

**Affiliations:** 1 IIHR—Hydroscience and Engineering, University of Iowa, Iowa City, USA; 2 Present Address: Department of Civil and Environmental Engineering, Princeton University, Princeton, USA; 3 Present Address: High Meadows Environmental Institute, Princeton University, Princeton, USA

**Keywords:** Projection and prediction, Hydrology, Water resources

Correction to: *Nature Communications* 10.1038/s41467-023-44415-4, published online 03 January 2024

The original version of this Article contained an error in discussed annual exceedance probabilities (AEP)s, which corresponded with Figs. 1 and 2, and Supplementary Figs. 2, 11, and 12. The correct version includes the correct AEP discussions. This has been corrected in both the PDF and HTML versions of the Article.

### Supplementary information

Updated Supplementary Information

