# Supplementary material for: Higher emissions scenarios lead to more extreme flooding in the United States
Source: Nat Commun. 2024 Jan 3;15:237. doi: 10.1038/s41467-023-44415-4 (PMC10764829; doi:10.1038/s41467-023-44415-4)
Supplement: Supplementary file 1 — Supplementary Information [file 41467_2023_44415_MOESM1_ESM.pdf]

Supplementary Information for

Higher emissions scenarios lead to more extreme flooding in the United States

Authors: Hanbeen Kim and Gabriele Villarini*

Affiliations: IIHR—Hydroscience and Engineering, University of Iowa, Iowa City, USA.

Current affiliation: Department of Civil and Environmental Engineering and High Meadows Environmental Institute, Princeton University, Princeton, USA.

*Corresponding author. Email: gvillari@princeton.edu

The PDF file includes:

Supplementary Figs. 1 to 13

Supplementary Table 1

Supplementary References

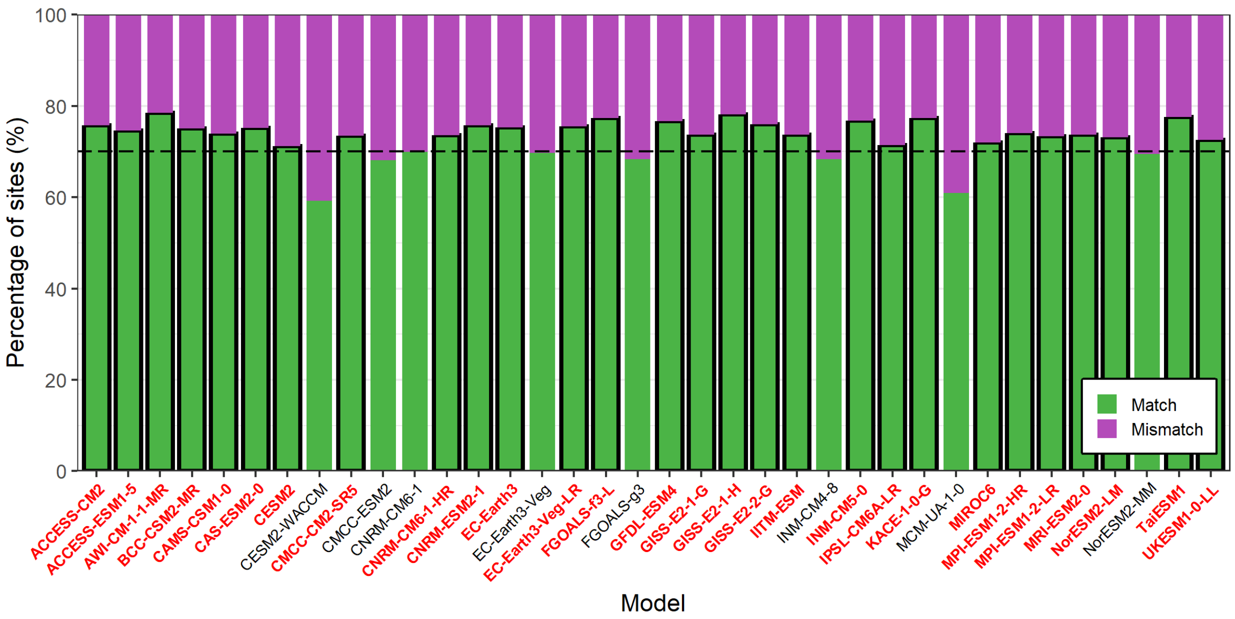

Supplementary Fig. 1. Evaluation of the suitability of GCMs in reproducing the trends in annual maximum discharge during the historical period. For 3,885 sites, Kendall's τ is computed over the period 1951–2014 from the median of the statistical model forced with observed or modeled precipitation and temperature. Results are classified into two groups: Match group) It includes sites where observed and modeled Kendall's τ values are not statistically significant, or significant at the 5% level and of the same sign; Mismatch group) it includes sites where observed (modeled) Kendall's τ values are not statistically significant, while modeled (observed) Kendall's τ values are significant at the 5% level. It also includes sites where observed and modeled Kendall's τ values are both significant at the 5% level but of opposite sign. The models in which the percentage of Match group is larger than 70% are highlighted in red text and with a bordered bar.

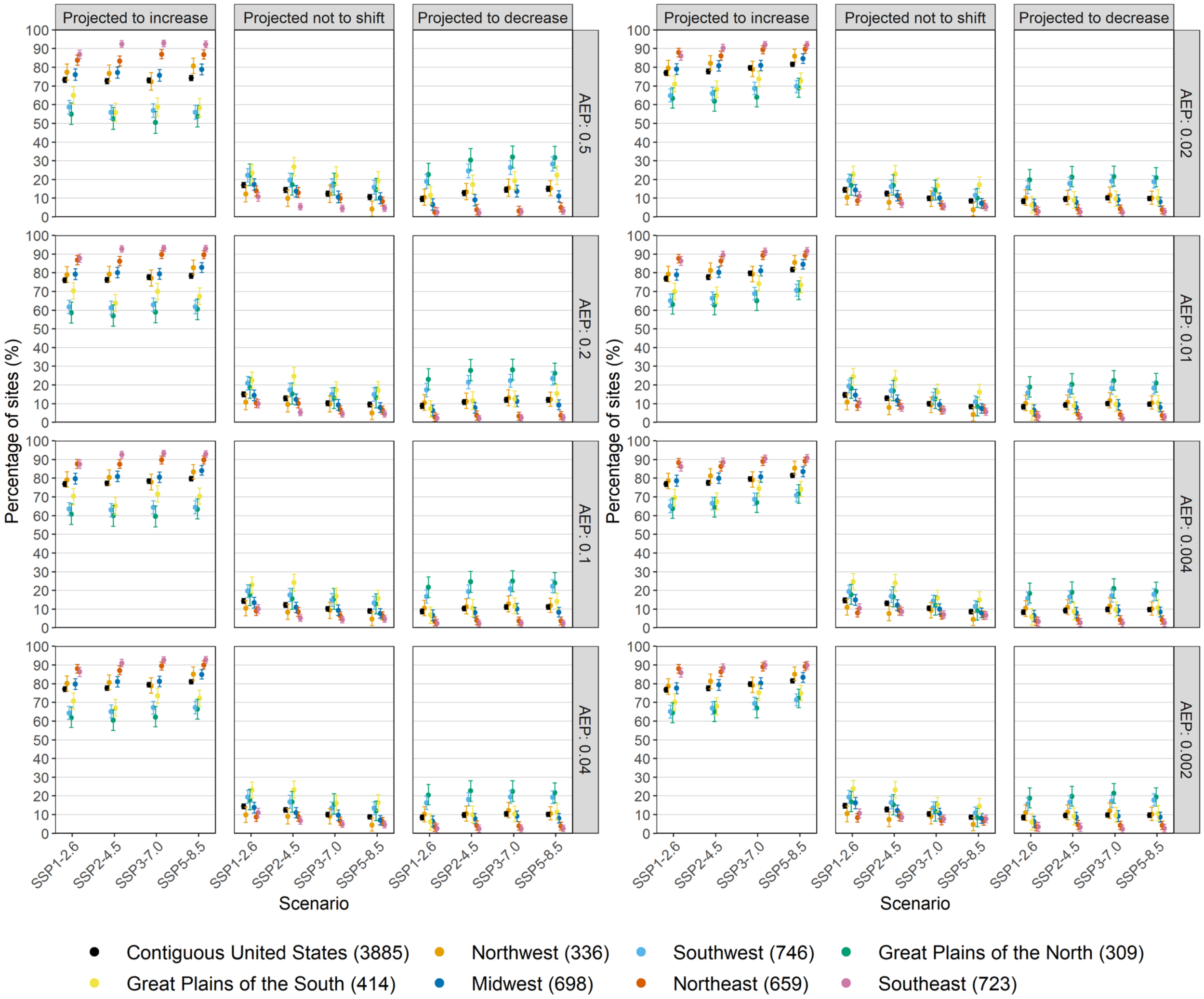

Supplementary Fig. 2. Projected shifts in the distribution of the annual maximum discharge during the historical (1985–2014) and future (2071–2100) periods for CONUS and seven subregions. The left (right) subplot shows the results for 0.5, 0.2, 0.1, and 0.04 (0.02, 0.01, 0.004, and 0.002) AEPs. In each subplot, the left (right) panels show the percentage of sites with a significant increasing (decreasing) shift in peak discharges for CONUS and its seven subregions (see inset map in Fig. 2 for the definition of regions) at the 5% level. The middle panel shows the percentage of sites where there is no significant shift in peak discharges at the 5% level. The error bars represent the 95% confidence intervals for multinomial proportions computed based on the Sison-Glaz method[1]. The numbers in the legend represent the number of streamgages within each region. Notice that jitters are added to the x-axis to improve readability.

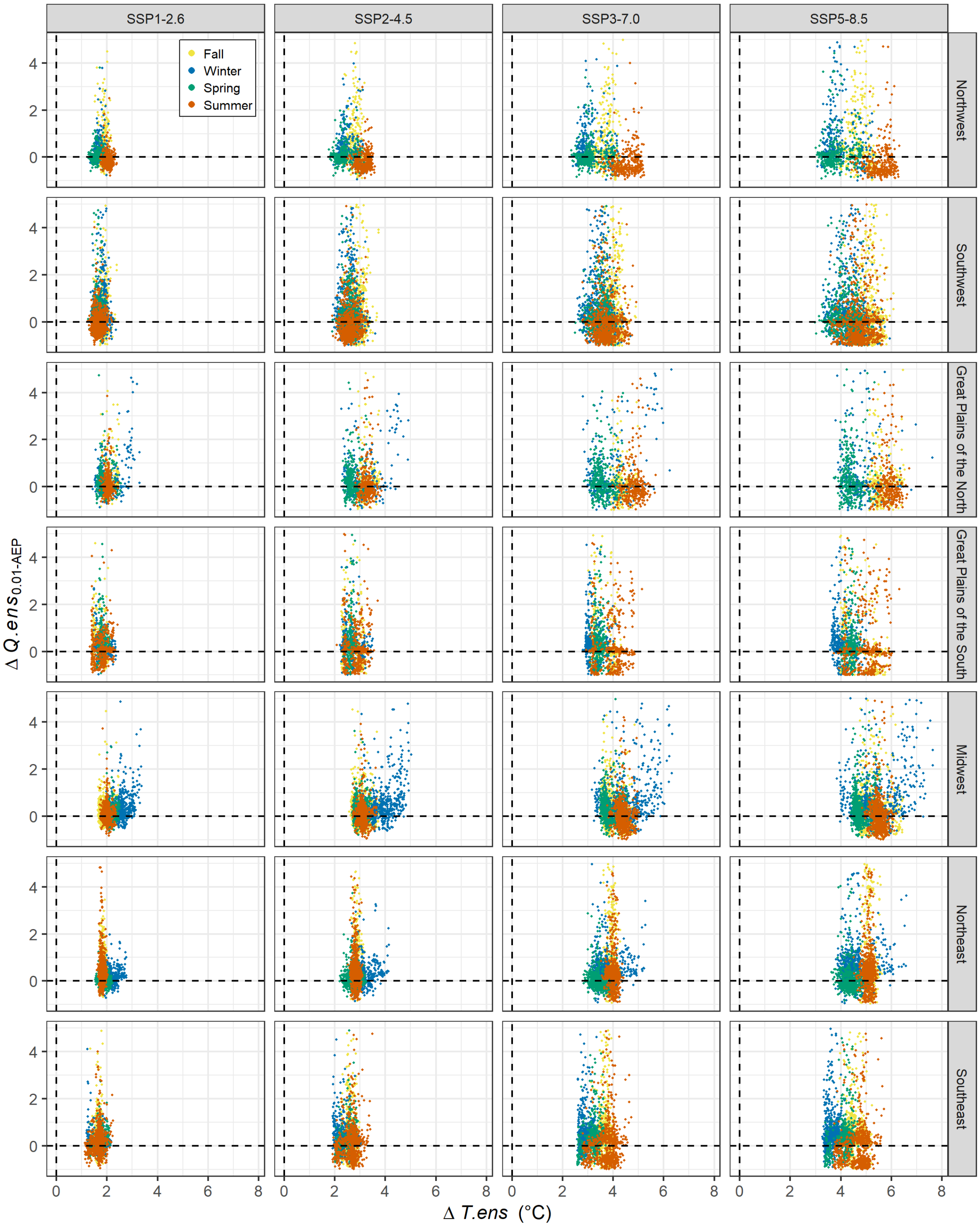

Supplementary Fig. 3. Relationship between the projected changes of the 0.01-AEP seasonal maximum discharge (Δ*Q.ens*$_{0.01\text{-}AEP}$) and temperature (Δ*T.ens*) during the historical (1985–2014) and future (2071–2100) periods. The ensemble mean of the GCMs' output is used to calculate the projected changes. In the case of seasonal maximum discharge, the relative changes are used to account for the differences in catchment size among sites.

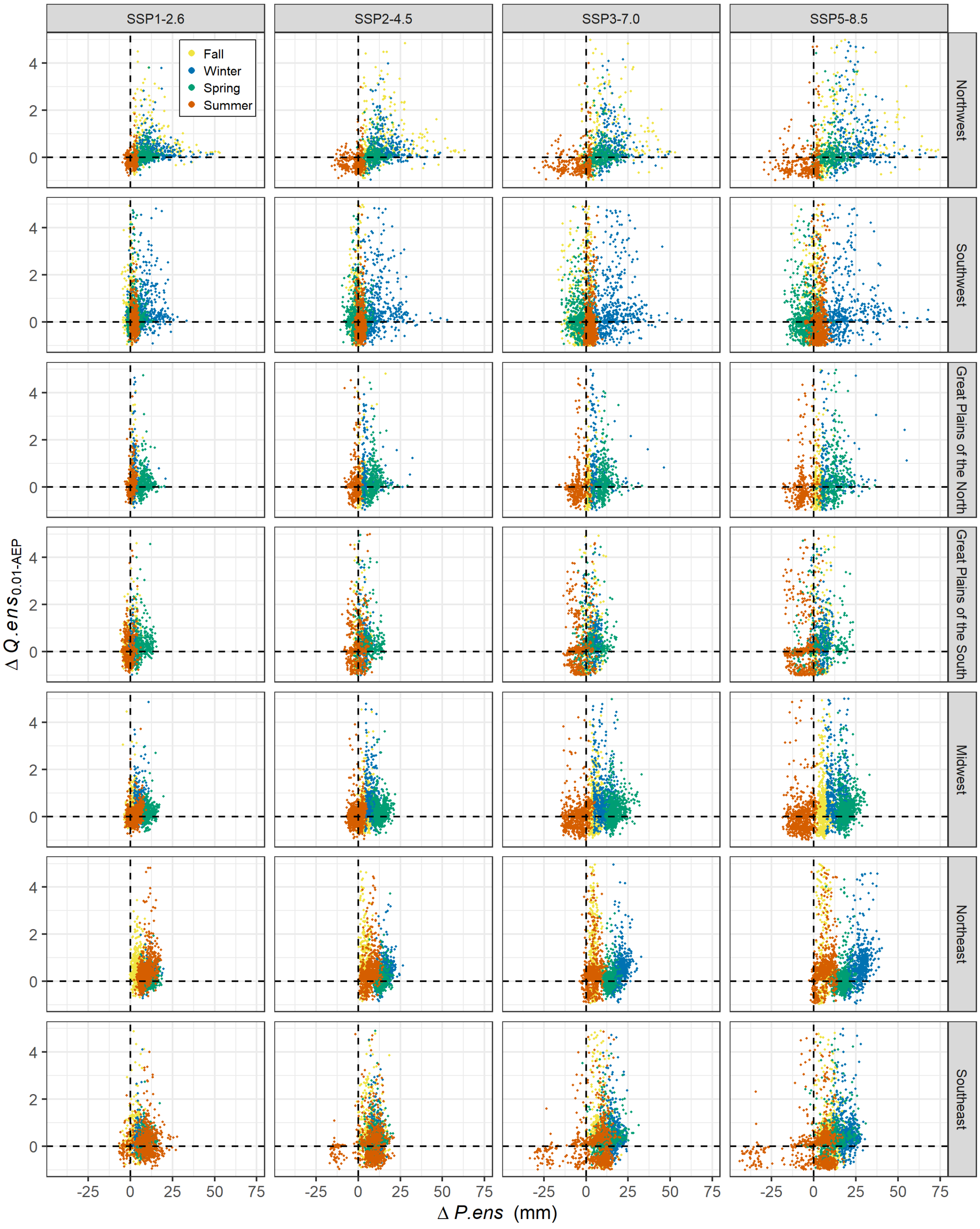

Supplementary Fig. 4. Relationship between the projected changes of the 0.01-AEP seasonal maximum discharge (Δ*Q.ens*$_{0.01\text{-}AEP}$) and precipitation (Δ*P.ens*) during the historical (1985–2014) and future (2071–2100) periods. The ensemble mean of the GCMs' output is used to calculate the projected changes. In the case of seasonal maximum discharge, the relative changes are used to account for the differences in catchment size among sites.

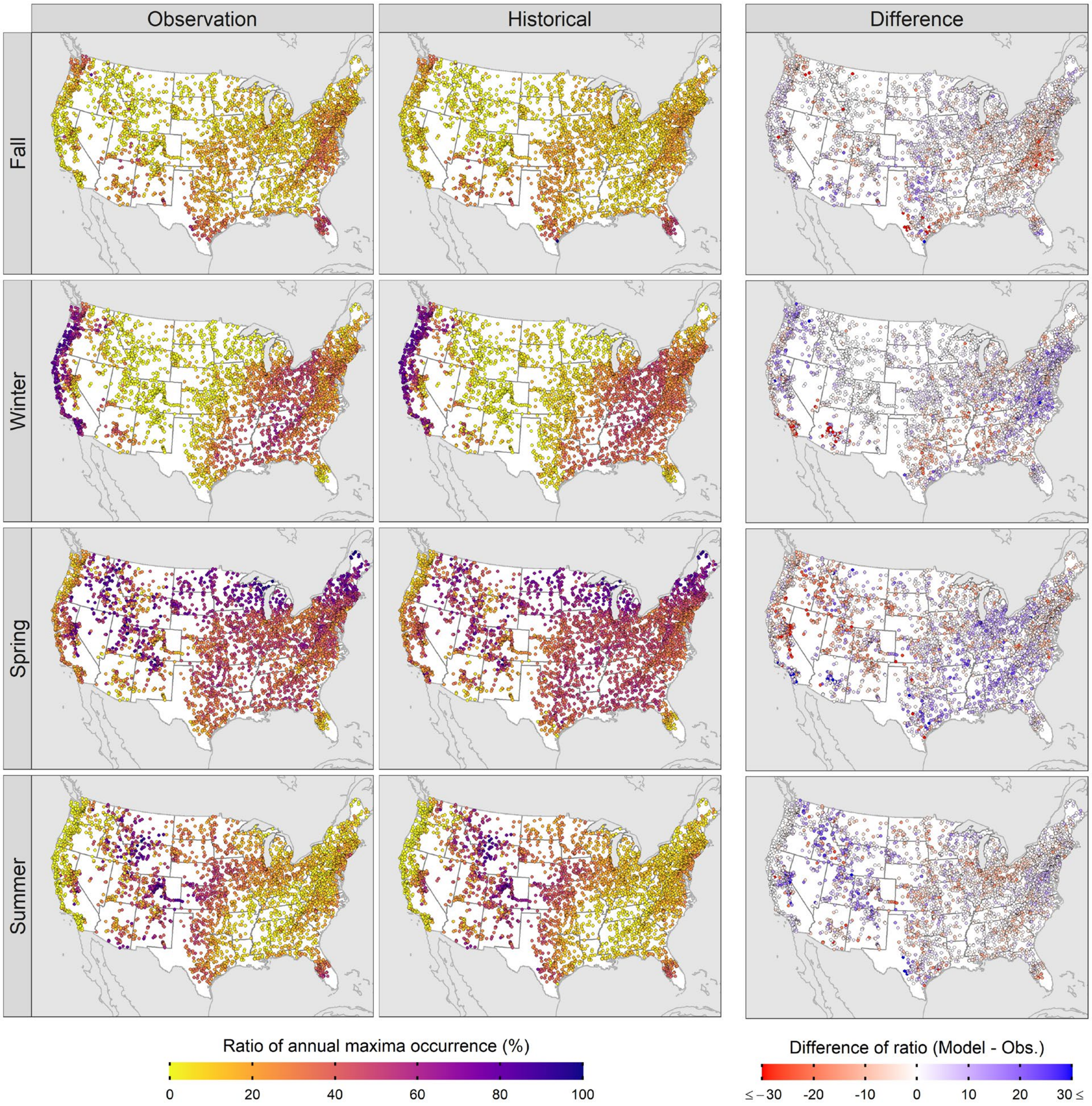

Supplementary Fig. 5. Assessment of the capability of the statistical models in reproducing the seasonality of annual maximum discharge occurrence during the historical period (1985–2014). The left (middle) column shows the percentage contribution of each season to the annual maximum based on the observations (the statistical model) for the historical period. The right column shows the difference between the left and right columns (i.e., model minus observation).

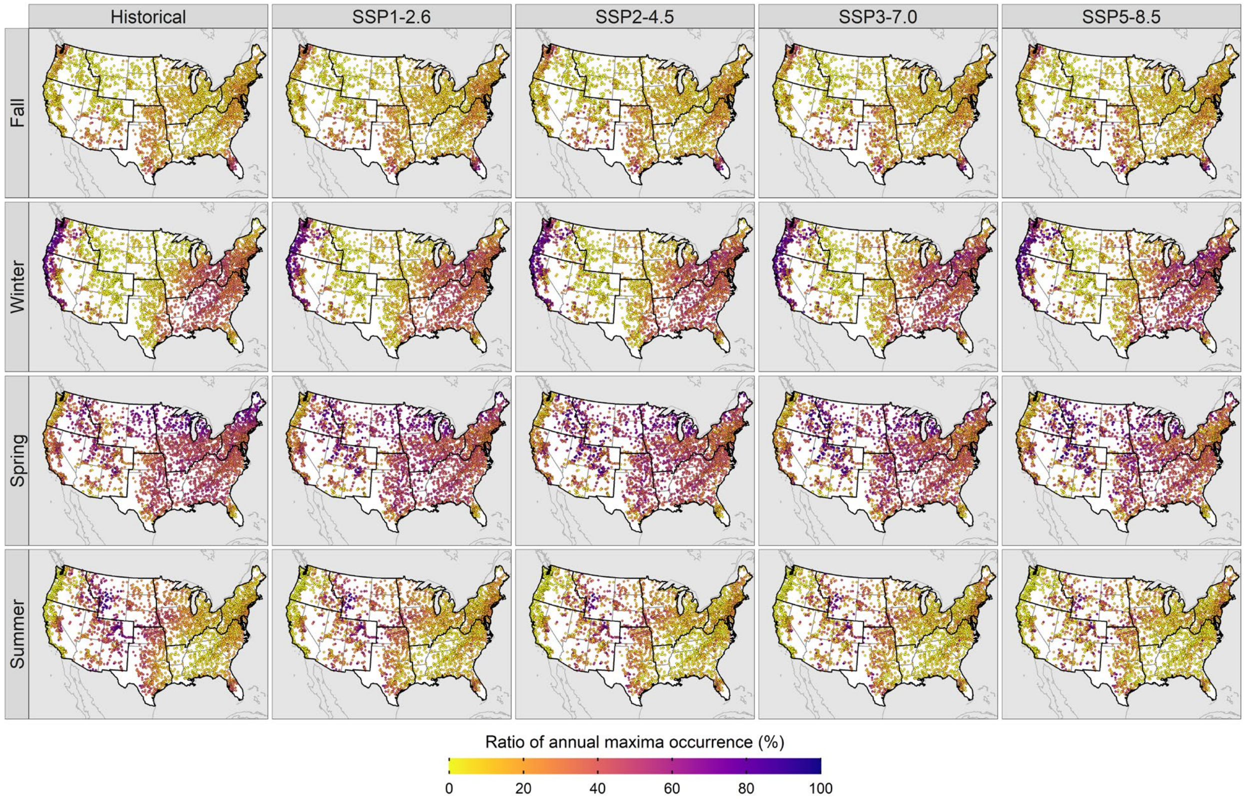

Supplementary Fig. 6. Seasonality of annual maximum discharge occurrence during the historical and future periods based on SSP1-2.6, SSP2-4.5, SSP3-7.0, and SSP5-8.5. Each map shows the percentage contribution of each season (organized along rows) to the annual maxima, stratified according to the historical (1985–2014) and future (2071–2100) periods (organized along columns).

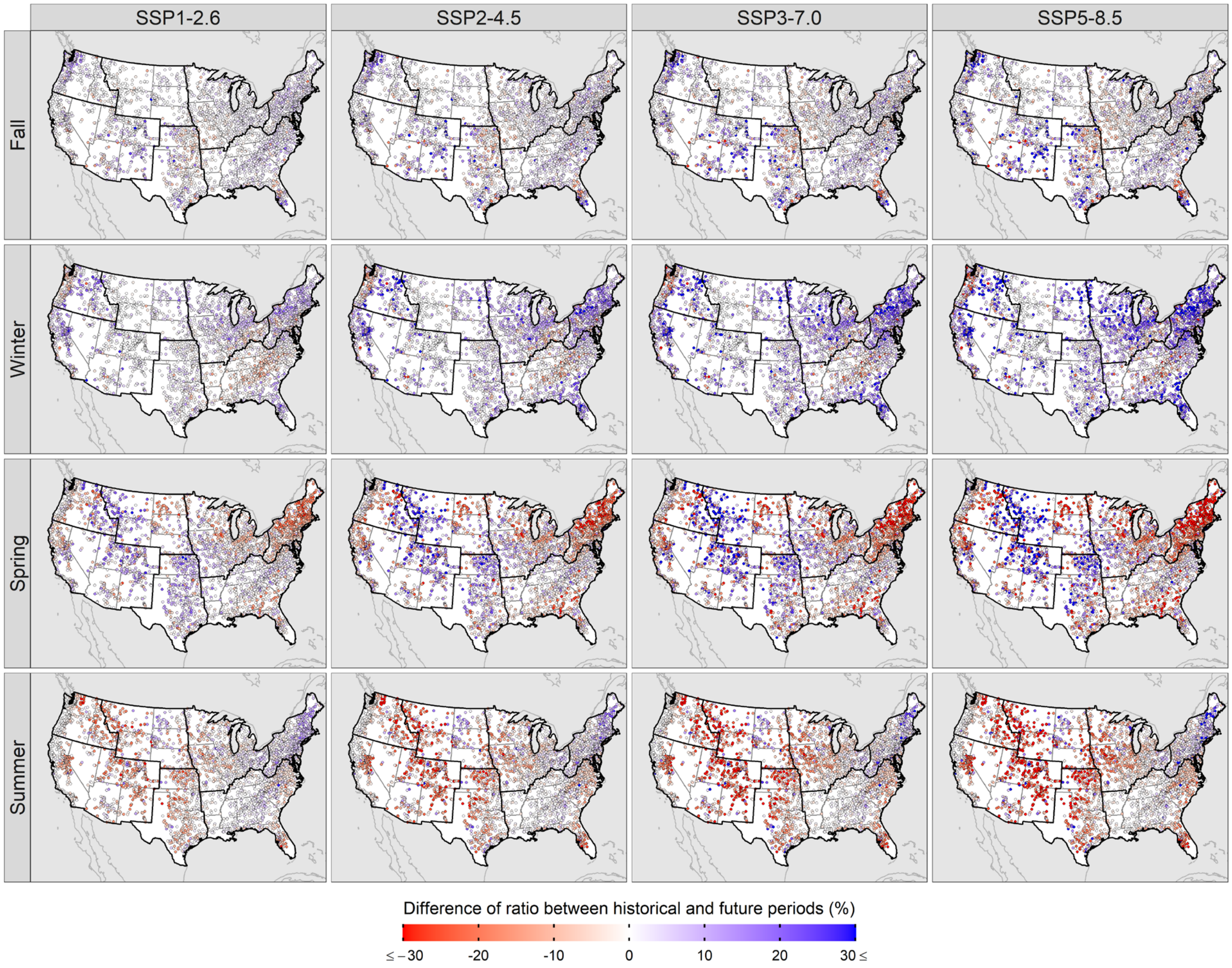

Supplementary Fig. 7. Projected changes in the seasonality of annual maximum discharge occurrence during the historical past and future period based on SSP1-2.6, SSP2-4.5, SSP3-7.0, and SSP5-8.5. The difference of seasonal ratio of the annual maximum discharge occurrence is obtained by subtracting the ratio for the historical period (1985–2014) from the ratio for the future period (2071–2100).

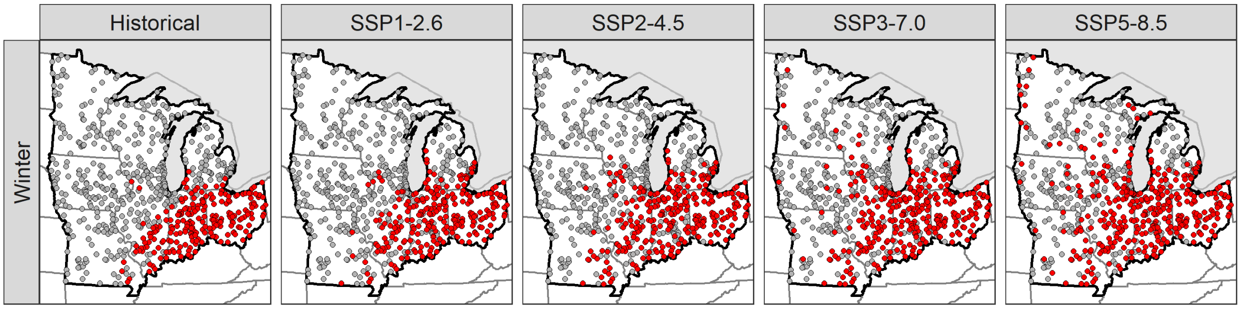

Supplementary Fig. 8. Projected changes in the percentage contribution of winter season to the annual maximum discharge during the historical (1985–2014) and future (2071–2100) periods for the Midwest. The red dots indicate the location of sites where the winter season accounts for at least 30% of annual maximum discharge occurrence, while the gray dots present the rest of the sites. The bold line presents the boundaries of the Midwest.

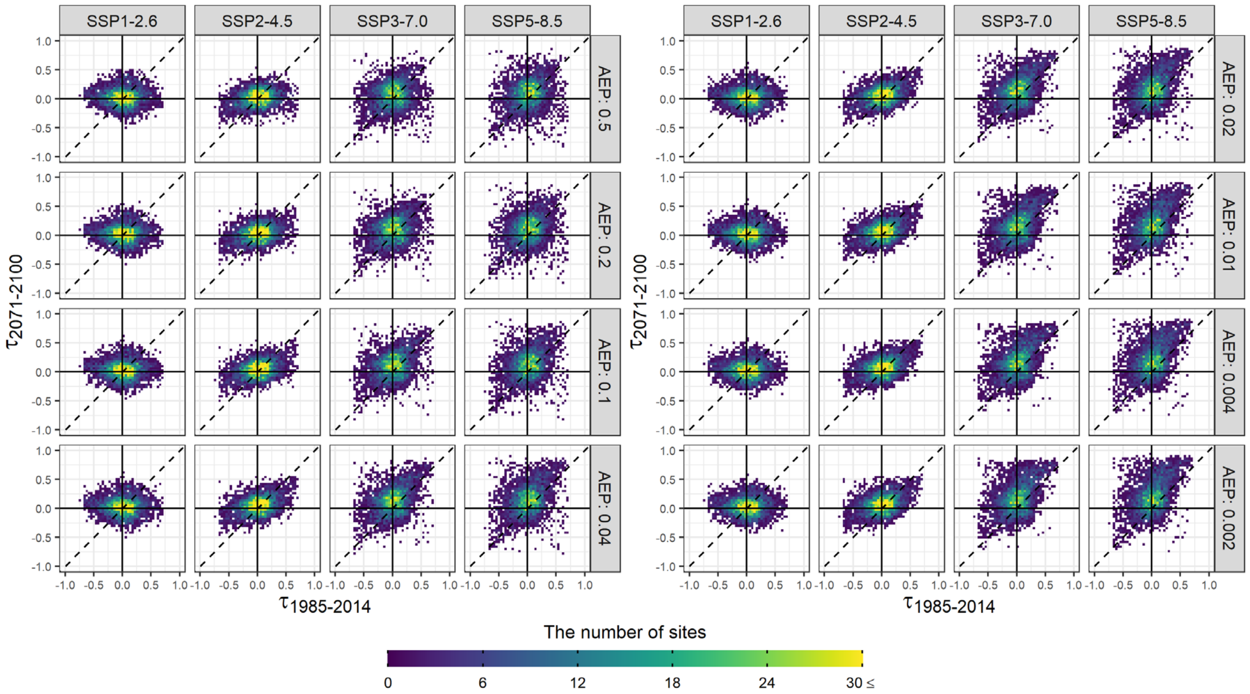

Supplementary Fig. 9. 2D histogram of trends in annual maximum discharge during the historical past and the future period. The Kendall's τ is computed for the historical (1985–2014) and future (2071–2100) periods for ensemble mean of annual maximum discharge. Each column (row) shows the results for each scenario (AEP).

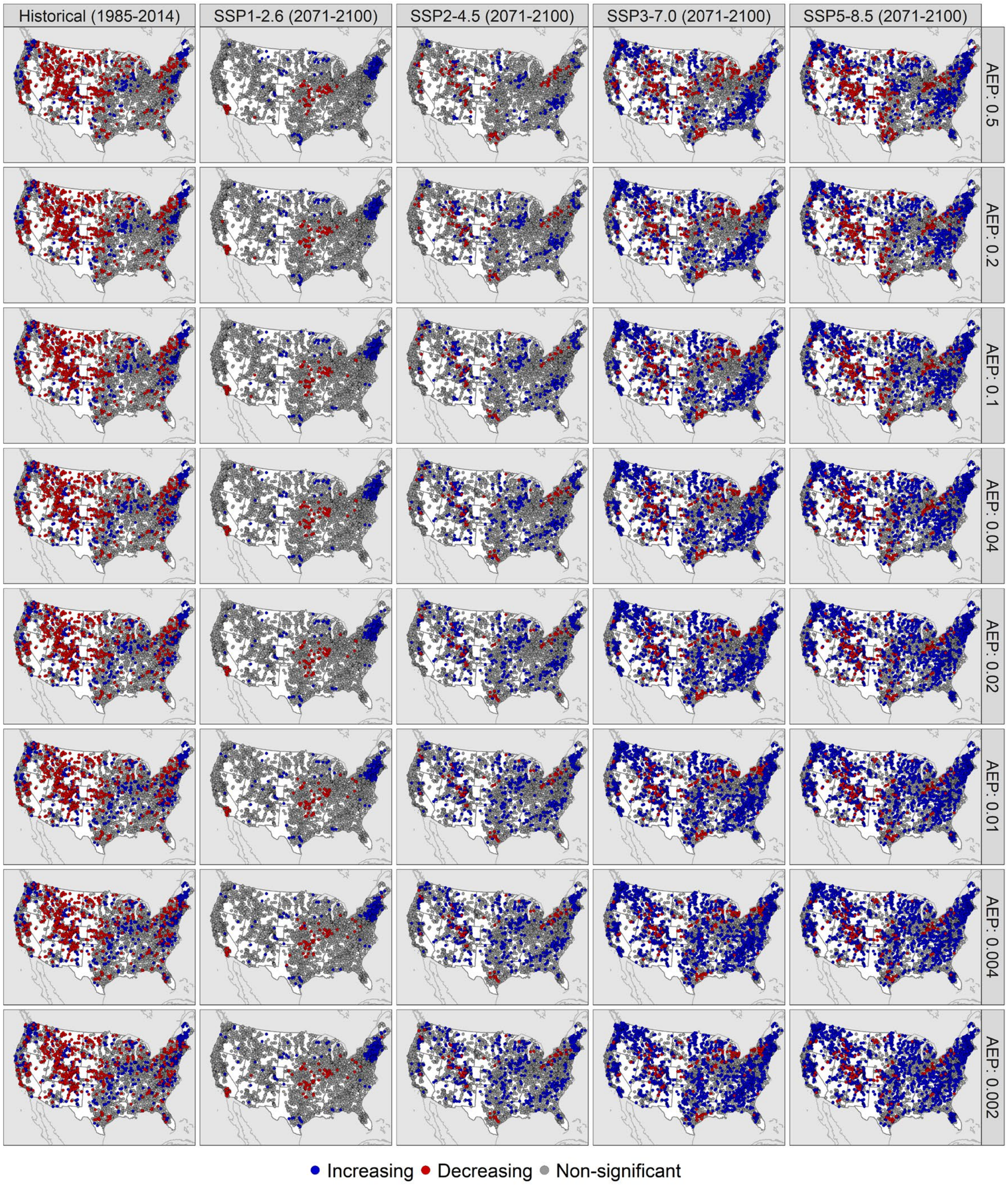

Supplementary Fig. 10. Comparison of trend signs in annual maximum discharge during the historical past and future period based on SSP1-2.6, SSP2-4.5, SSP3-7.0, and SSP5-8.5. The Kendall's t is computed for the historical (1986–2014) and future (2071–2100) periods. Blue (red) circles indicate the sites with a significant increasing (decreasing) trend in ensemble mean of peak discharges at a 5% significance level, while the gray circles indicate the sites with no significant trend. Each row shows the results for each AEP.

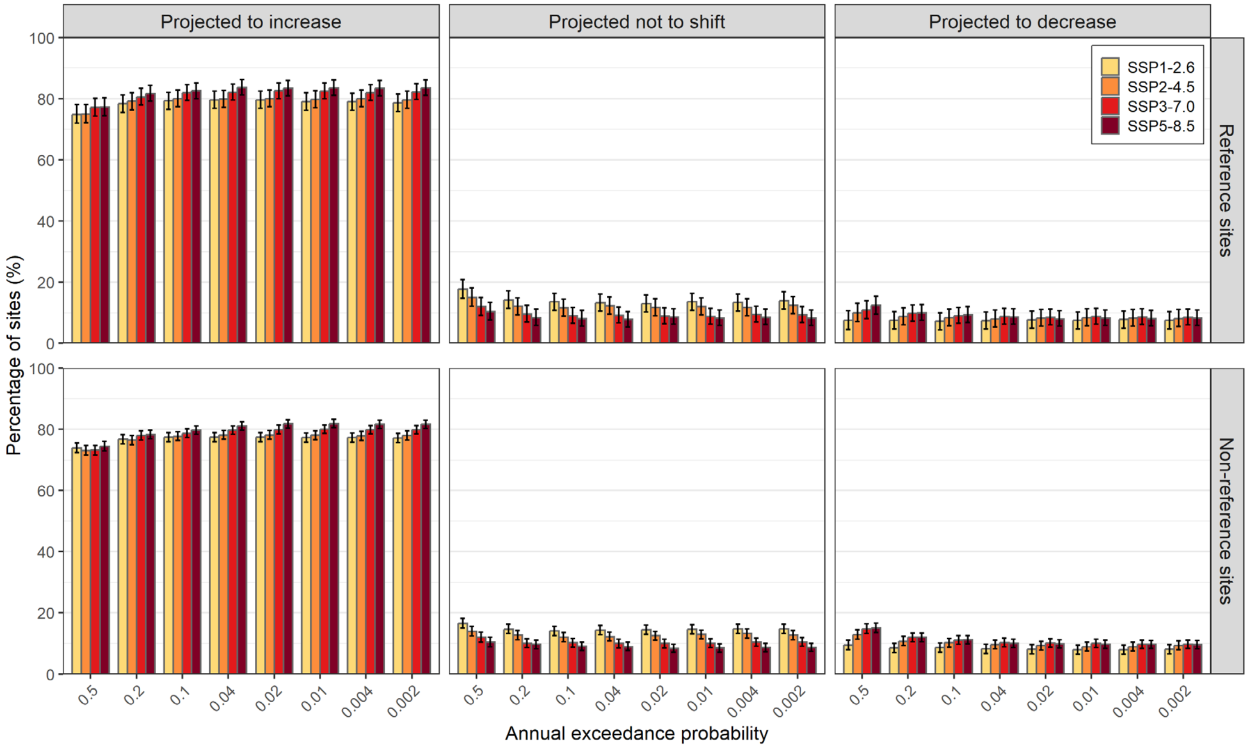

Supplementary Fig. 11. Projected shifts in the distribution of the annual maximum discharge during the historical (1985–2014) and future (2071–2100) periods for CONUS according to the GAGES-II classification. The top (bottom) row shows the results for reference (non-reference) sites. The left (right) panels show the percentage of sites with a significant increasing (decreasing) shift in peak discharges for CONUS at the 5% level. The middle panels show the percentage of sites where there is no significant shift in peak discharges at the 5% level. In each panel, redder bars indicate higher emission scenarios from SSP1-2.6 to SSP 5-8.5. The error bars represent the 95% confidence intervals for multinomial proportions computed based on the Sison-Glaz method[1].

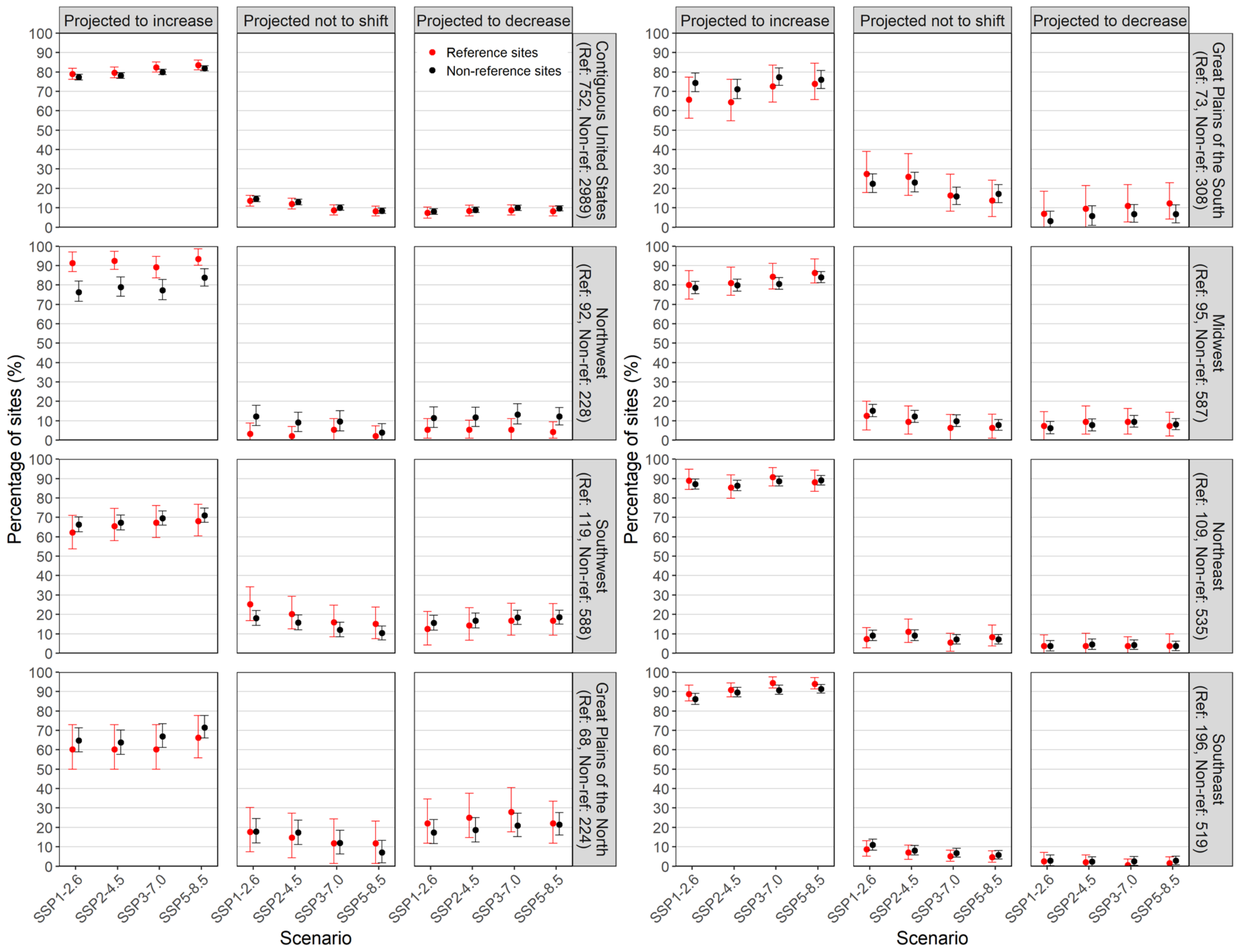

Supplementary Fig. 12. Projected shifts in the distribution of the 0.01-AEP discharge during the historical (1985–2014) and future (2071–2100) periods for CONUS and seven subregions according to the GAGES-II classification. The left (right) subplot shows the results for CONUS, Northwest, Southwest, and Great Plains of the North (Great Plains of the South, Midwest, Northeast, and Southeast). In each subplot, the left (right) panels show the percentage of sites with a 5%-significant increasing (decreasing) shift in peak discharges for reference and non-reference sites. The middle panels show the percentage of sites where there is no significant shift in peak discharges at the 5% level. The error bars represent the 95% confidence intervals for multinomial proportions computed based on the Sison-Glaz method[1]. The numbers under the region name represent the number of reference and non-reference streamgages within each region.

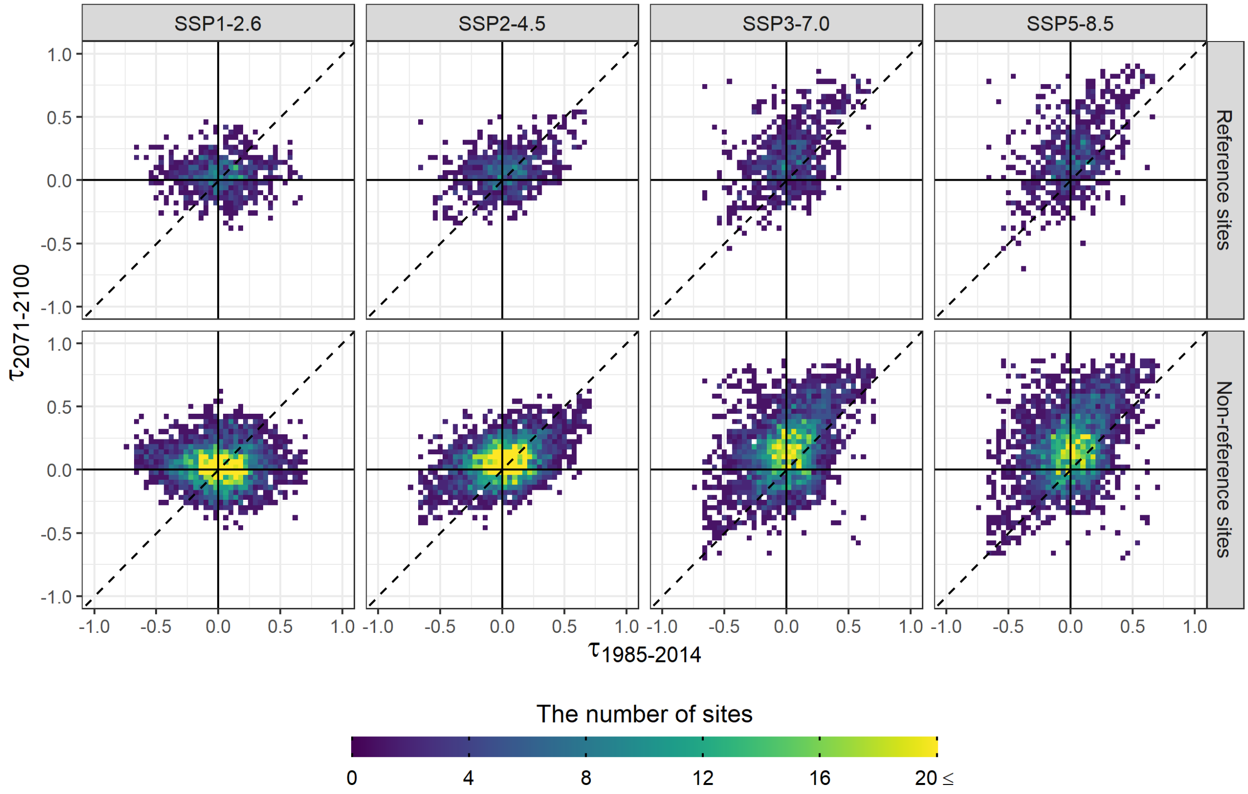

Supplementary Fig. 13. 2D histogram of trends in 0.01-AEP discharge during the historical past and the future period according to the GAGES-II classification. The Kendall's τ is computed for the historical (1985–2014) and future (2071–2100) periods for ensemble mean of 0.01-AEP discharge. The top (bottom) row shows the results for reference (non-reference) sites.

Supplementary Table 1. Information about the 36 GCMs initially considered in this study. A subset of 28 GCMs are used in the analyses based on their performance in reproducing the historical trends (identified by '*').

Model	Spatial resolution	Variant
ACCESS-CM2*	250km	r1i1p1f1
ACCESS-ESM1-5*	250km	r1i1p1f1
AWI-CM-1-1-MR*	100km	r1i1p1f1
BCC-CSM2-MR*	100km	r1i1p1f1
CAMS-CSM1-0*	100km	r1i1p1f1
CAS-ESM2-0*	100km	r1i1p1f1
CESM2*	100km	r4i1p1f1
CESM2-WACCM	100km	r1i1p1f1
CMCC-CM2-SR5*	100km	r1i1p1f1
CMCC-ESM2	100km	r1i1p1f1
CNRM-CM6-1	250km	r1i1p1f2
CNRM-CM6-1-HR*	50km	r1i1p1f2
CNRM-ESM2-1*	250km	r1i1p1f2
EC-Earth3*	100km	r1i1p1f1
EC-Earth3-Veg	100km	r1i1p1f1
EC-Earth3-Veg-LR*	250km	r1i1p1f1
FGOALS-f3-L*	100km	r1i1p1f1
FGOALS-g3	250km	r1i1p1f1
GFDL-ESM4*	100km	r1i1p1f1
GISS-E2-1-G*	250km	r1i1p1f2
GISS-E2-1-H*	250km	r1i1p1f2
GISS-E2-2-G*	250km	r1i1p3f1
IITM-ESM*	250km	r1i1p1f1
INM-CM4-8	100km	r1i1p1f1
INM-CM5-0*	100km	r1i1p1f1
IPSL-CM6A-LR*	250km	r1i1p1f1
KACE-1-0-G*	250km	r1i1p1f1
MCM-UA-1-0	250km	r1i1p1f2
MIROC6*	250km	r1i1p1f1
MPI-ESM1-2-HR*	100km	r1i1p1f1
MPI-ESM1-2-LR*	250km	r1i1p1f1
MRI-ESM2-0*	100km	r1i1p1f1
NorESM2-LM*	250km	r1i1p1f1
NorESM2-MM	100km	r1i1p1f1
TaiESM1*	100km	r1i1p1f1
UKESM1-0-LL*	250km	r1i1p1f2

Supplementary References

1 Sison, C. P. & Glaz, J. Simultaneous Confidence-Intervals and Sample-Size Determination for Multinomial Proportions. *J Am Stat Assoc* **90**, 366-369 (1995). https://doi.org:Doi 10.2307/2291162
